# Comprehensive meta-analysis of anti-BCMA chimeric antigen receptor T-cell therapy in relapsed or refractory multiple myeloma

**DOI:** 10.1080/07853890.2021.1970218

**Published:** 2021-08-30

**Authors:** Lina Zhang, Xuxing Shen, Wenjun Yu, Jing Li, Jue Zhang, Run Zhang, Jianyong Li, Lijuan Chen

**Affiliations:** a Department of Hematology, The First Affiliated Hospital of Nanjing Medical University, Jiangsu Province Hospital, Collaborative Innovation Center for Cancer Personalized Medicine, Nanjing, China; b Department of Geriatric Medicine, Geriatric Hospital of Nanjing Medical University, Jiangsu Province Geriatric Institute, Nanjing, China

**Keywords:** Chimeric antigen receptor T-cell therapy, multiple myeloma, efficacy, safety

## Abstract

**Background:**

Chimeric antigen receptor (CAR) T-cell therapy shows impressive results in clinical trials. We conducted a meta-analysis based on the most recent data to systematically describe the efficacy and safety of anti-BCMA CAR T therapy for patients with relapsed or refractory multiple myeloma (R/R MM).

**Methods:**

PubMed, Embase, Web of Science, Cochrane library, ClinicalTrials.gov, China Biology Medicine disc (CBM disc) and Wanfang Data were searched on 8 November 2020. Registration number of PROSPERO was CRD42020219127.

**Results:**

From 763 articles, we identified 22 appropriate studies with 681 patients. The pooled overall response rate (ORR) was 85.2% (95%CI 0.797–0.910), complete response rate (CRR) was 47.0% (95%CI 0.378–0.583), and minimal residual disease (MRD) negativity rate was 97.8% (95%CI 0.935–1.022). The pooled incidence of grade 3–4 cytokine release syndrome was 6.6% (95%CI 0.036–0.096) and neurotoxicity was 2.2% (95%CI 0.006–0.038). The median progression-free survival (PFS) was 14.0 months and median overall survival (OS) was 24.0 months. Subgroup analysis showed dual epitope-binding CAR T cells achieved the best therapy outcomes and humanized CAR T cells had the best safety profile. Patients who were older, heavily pre-treated or received lower dose of CAR T cells had worse ORR. There was no significant difference in ORR, CRR and PFS between patients with and without high-risk cytogenetic features. The PFS and CRR of non-extramedullary disease (EMD) group was superior to those of EMD group.

**Conclusion:**

Anti-BCMA CAR T therapy is effective and safe for patients with R/R MM. It can improve the prognosis of patients with high-risk cytogenetic features while the prognosis of patients with EMD remains poor. Moreover, patients are likely to benefit from an earlier use of CAR T therapy and human-derived CAR T cells have obvious advantages based on the existing data.

## 1. Introduction

Multiple myeloma (MM) is a tumour of plasma cells whose incidence rate ranks second in hematological malignancies [1]. It remains an incurable disease because most patients experience relapse and become refractory to chemotherapeutic agents [2]. Over the past decade, chimeric antigen receptor (CAR) T-cell therapy has brought hope to patients with relapsed or refractory multiple myeloma (R/R MM) [3–5]. CAR T therapy is a novel immunotherapy for tumors wherein T cells are genetically modified to express chimeric antigen receptors and then eliminate the tumour cells bearing the same antigens [6,7]. During the production of CAR T therapy, T cells can be harvested from a patient (autologous) or a healthy donor (allogeneic) [8,9]. The majority of clinical trials for MM are currently testing the efficacy and safety of autologous CAR T [10]. Therefore, in this paper, we focus on the data of autologous CAR T.

On the other hand, several antigens have been used as targets for MM in both preclinical and clinical studies, including B cell maturation antigen (BCMA), CD19, CD138, immunoglobulin light chains, signalling lymphocytic activation molecule 7 (SLAM7), and so forth [3,10,11]. Of these, anti-BCMA CAR T therapy is the most commonly studied in R/R MM [10,12]. The excellent efficacy and safety of anti-BCMA CAR T have been reported by numerous phase 1/2 clinical trials. Even those patients who experience multiple relapses after multimodal therapy (chemotherapy, monoclonal antibody and autologous stem cell transplantation) can achieve remission [3,5,10,12]. Adverse effects associated with CAR T include cytokine release syndrome (CRS) and neurotoxicity, which rarely cause the death of patients [13,14].

A previous meta-analysis showed the pooled overall response rate (ORR) of anti-BCMA CAR T therapy for R/R MM was 82%, and severe CRS and neurotoxicity (grade 3–4) occurred only in 15% and 18% [15]. However, more clinical data are available as the number of clinical trials increases. Moreover, there are still some issues of CAR T therapy which remain unclear. For example, whether it is safe and effective also in elderly patients? Whether this new treatment could improve outcomes of MM patients with high risk factors? What factors are associated with its efficacy and toxicity? To provide a comprehensive overview of anti-BCMA CAR T therapy in R/R MM, we conducted a meta-analysis based on the most recent data by involving 681 patients from 22 studies.

## 2. Methods

The methods adopted for this systematic review and meta-analysis were compliant with the Preferred Reporting Items for Systematic Review and Meta-Analysis (PRISMA) guidelines. The protocol for this meta-analysis has been registered in PROSPERO (registration number: CRD42020219127).

### 2.1. Inclusion and exclusion criteria

#### Study type

Single-arm prospective studies were eligible for inclusion, which could be single center or multi-center.

#### Population

Patients with R/R MM were enrolled.

#### Intervention

Patients received anti-BCMA CAR T-cell infusion were enrolled.

#### Outcomes

Efficacy and/or safety outcome measures were involved, including the overall response rate (ORR), complete response rate (CRR), minimal residual disease (MRD) negativity, progression-free survival (PFS), overall survival (OS), and adverse events (AEs). AEs were graded following National Cancer Institute Common Terminology Criteria for Adverse Events (NCI-CTCAE v. 4.03). The CRS and neurotoxicity grades were determined by the criteria reported by the CARTOX working group [14]. ORR included partial response (PR), very good partial response (VGPR), complete response (CR) and stringent complete response (sCR). CRR included CR and sCR.

#### Exclusion criteria

Case reports, dual-target CAR T studies and studies using cocktail strategy (combining CAR T cells with different targets in a cocktail infusion) were excluded.

### 2.2. Search strategy

We searched PubMed, Embase, Web of Science, Cochrane library, ClinicalTrials.gov, China Biology Medicine disc (CBM disc) and Wanfang Data on 21 April 2021. Abstracts from American Society of Haematology (ASH) were also scanned on 9 December 2020 to ensure data integrity. The retrieval strategy was based on a combined use of subject words and free words, and adjusted according to different databases (see Supplementary materials for an example full search strategy).

### 2.3. Article selection and data extraction

EndNote X9 software was used for article selection. Three authors (L.Z., W.Y. and X.S.) independently screened the titles and abstracts. All potentially relevant articles were investigated by reading the full text. Disagreements in the selection of studies were resolved by a fourth author (L.C.). Data extraction was also performed independently by three authors (L.Z., W.Y. and X.S.) using Excel (Microsoft 2010). Disputes were resolved through discussion between authors, with a fourth author (L.C.) adjudication if necessary. The following data were collected: authors, year of publication, median age of patients, prior lines of therapy, product names and structural characteristics of CAR T-cells, the dose of infused CAR^+^ T cells, number of patients, fluorescence *in situ* hybridization (FISH) results of patients, BCMA expression of tumour cells, extramedullary disease (EMD) of patients, efficacy outcome measures and safety outcome measures.

### 2.4. Assessment of study quality and publication bias

The methodological quality was evaluated using Methodological Index for Non-Randomized Studies (MINORS) scale [16]. Given that all the studies had no control group, only 8 items of MINORS were used and the maximum score of each study was 16. Publication bias was evaluated using funnel plots and confirmed by Egger’s and Begg’s test.

### 2.5. Statistical analysis

Meta-analysis was carried out using Stata software (version 16.0). All statistical tests were two-sided, and *P* ≤ .05 was considered statistically significant. Heterogeneity was evaluated using the I^2^ test and Q test; I^2^>50% and *p* < .1 indicated heterogeneity across studies. I^2^ < 25%, 25%–50%, and >50% were considered as low, moderate, and high heterogeneity, respectively. Meta-regression was performed to study the causes of heterogeneity. A fixed-effect model was used when there was low heterogeneity, while a random-effect model was applied when there was obvious heterogeneity. Effects were expressed as pooled odds ratios (event rates) with 95% confidence intervals (CI). The confidence interval was calculated using the Clopper–Pearson formula. PFS and OS curves were assessed using GraphPad Prism software (version 5.0) with the Kaplan–Meier method and compared by the log-rank test.

## 3. Results

### 3.1. Results of the search

**Figure 1.**
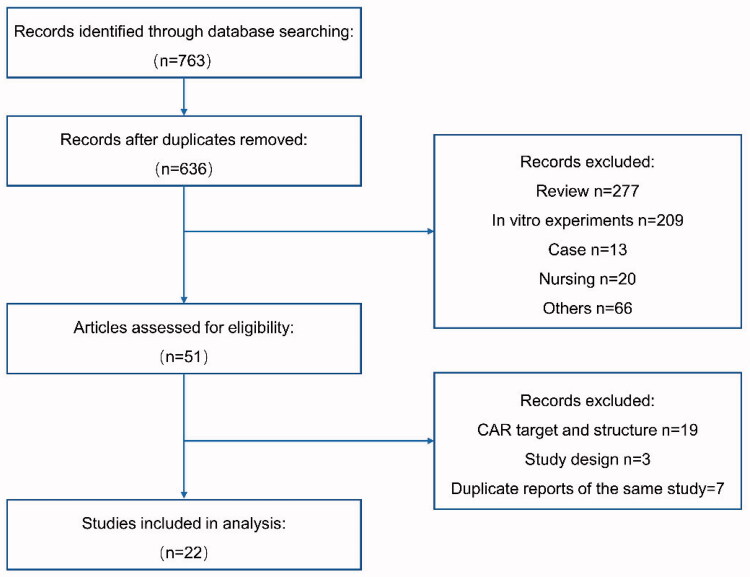
Flow chart of literature screening process.

A total of 763 articles were retrieved from databases search. After removal of duplicates, and screening of titles and abstracts, 51 records were left for full-text evaluation. After reading the full text, a total of 22 documents were selected for full analysis. For the same study, only the latest results were selected. Figure 1 showed the flow chart of the selection process. The clinical characteristics and quality assessment of the included studies were listed in Table 1. For the 22 non-comparative studies, the median MINORS score was 12 (range, 11–13), indicating fair-quality evidence (maximum score was 16).

**Table 1.** Characteristics of the selected studies.

Study	Production name	Costimulatory molecules	ScFv origin	Median age (years)	Lines of prior regimens	No.	CAR^+^ T infused	ORR (%)	CRR (%)	MRD negativity (%)	Severe* CRS (%)	Severe* neurotoxicity (%)	MINORS score
Alsina 2020 [17]	bb21217	4-1BB	Murine	62	6	46	150, 300 or 450 × 10^6^	54.5	18.2	NA	4.3	6.5	13
An 2020 [18]	C-CAR088	4-1BB	Human	60	4	21	1, 3, 4.5–6 × 10^6^/kg	95.2	28.6	NA	8.3	0.0	12
Brudno 2018 [19]	CAR-BCMA	CD28	Murine	56.5	9.5	16	9 × 10^6^/kg	81.3	12.5	100.0	37.5	6.3	12
Chen 2020 [20]	CT053	4-1BB	Human	54	6	12	100–150 × 10^6^	100.0	41.7	100.0	0.0	0.0	12
Cohen 2019 [21]	CART-BCMA	4-1BB	Human	58	7	25	100–500 or 10–50 × 10^6^	48.0	8.0	NA	32.0	12	12
Costello 2020 [22]	P-BCMA-101	4-1BB	Human	60	7	43	A median dose of 0.75 × 10^6^/kg	55.9	NA	NA	2.3	0.0	12
Frigault 2020 [23]	CART- Ddbcma	4-1BB	Unknown	73.5	5	4	100 × 10^6^±20%	100.0	100.0	100.0	0.0	0.0	13
Fu 2019 [24]	HRAIN	4-1BB	Unknown	NA	NA	44	9 × 10^6^/kg	79.6	40.9	NA	6.8	NA	11
Green 2018 [25]	NA	4-1BB	Human	63	8	7	50 or 150 × 10^6^	100.0	NA	NA	0.0	0.0	12
Han 2018 [26]	#3 CART-BCMA	4-1BB	Human heavy-chain-only	57	12.5	4	5 or 10 × 10^6^/kg	100.0	25.0	NA	25.0	25.0	11
Hao 2020 [27]	CT053	4-1BB	Human	60.1	4.5	24	50, 100, 150 or 180 × 10^6^	87.5	79.2	NA	0.0	4.2	12
Kumar 2020 [28]	CT053	4-1BB	Human	59	6	14	150–180 or 250–300 × 10^6^	100.0	40.0	91.7	0.0	0.0	12
Li 2018 [29]	BRD015	CD28	Murine	55	4	28	A median dose of 11.2 × 10^6^/kg	89.3	NA	70.6	14.3	NA	12
Lin 2020 [30]	bb2121	4-1BB	Murine	61	NA	62	50, 150, 450 or 800 × 10^6^	75.8	38.7	81.1	6.5	3.2	12
Madduri 2020 [31]	LCAR-B38M	4-1BB	Llama heavy-chain-only	61	6	97	0.5–1.0 × 10^6^/kg	94.8	55.7	94.2	4.1	10.3	13
Mailankody 2018 [32]	JCARH125	4-1BB	Human	53	10	8	50 or 150 × 10^6^	87.5	37.5	NA	0.0	12.5	12
Mailankody 2018-2 [33]	MCARH171	4-1BB	Human	NA	6	11	72, 137, 475 or 818 × 10^6^	63.6	NA	NA	18.2	0.0	12
Mikkilineni 2019 [34]	FHVH33	4-1BB	Human heavy-chain-only	63	6	12	0.75, 1.5 or 3 × 10^6^/kg	83.3	16.7	NA	8.3	8.3	13
Munshi 2021 [35]	bb2121	4-1BB	Murine	61	6	128	150–450 × 10^6^	72.7	32.8	100.0	5.5	3.1	13
Wang 2021 [36]	CT103A	4-1BB	Human	53.5	4	18	1, 3 or 6 × 10^6^/kg	100.0	72.2	44.4	27.8	0.0	12
Xu 2019 [37]	LCAR-B38M	4-1BB	Llama heavy-chain-only	56	4	17	0.21–1.52 × 10^6^/kg	88.2	76.5	NA	41.2	NA	12
Zhao 2018 [38]	LCAR-B38M	4-1BB	Llama heavy-chain-only	54	3	57	A median dose of 0.5 × 10^6^/kg	87.7	68.4	72.0	7.0	0.0	12

ScFv: single-chain variable fragment; No: number of participants; ORR: overall response rate; CRR: complete response rate; MRD: minimal residual disease; CRS: cytokine release syndrome; NA: not available.

*Severe CRS and severe neurotoxicity: grade 3–4 CRS and grade 3–4 neurotoxicity.

### 3.2. Efficacy

#### 3.2.1. Pooled rates

**Figure 2.**
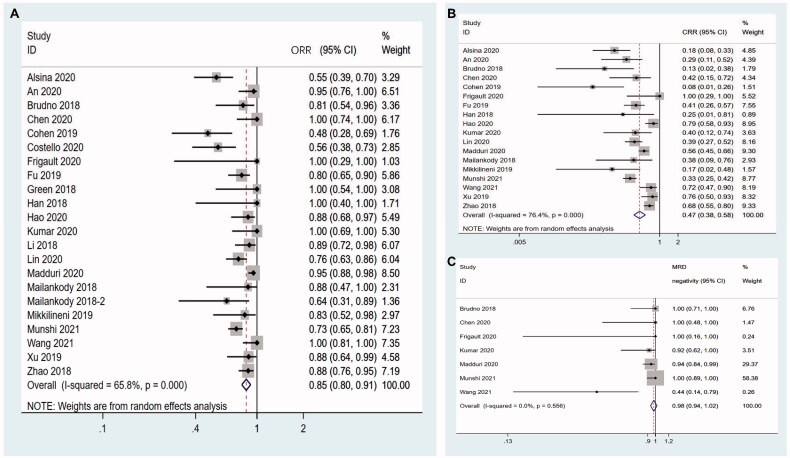
Pooled rates of ORR, CRR and MRD negativity. (A) The pooled ORR is 85.2%. (B) The pooled CRR is 47.0%. (C) The pooled rate of MRD negativity is 97.8%.

All twenty-two studies reported ORR and the pooled ORR was 85.2% (95%CI 0.797–0.910). As there was marked heterogeneity (I^2^ = 65.8%, *p* < .001), the random-effects model was selected (Figure 2(A)). Eighteen studies had CRR data and the pooled CRR was 47.0% (95%CI 0.378–0.583). The random-effects model was used as there was significant heterogeneity between studies (I^2^ = 76.4%, *p* < .001) (Figure 2(B)). In ten studies, MRD was detected in patients who achieved remission. However, only seven out of ten articles used next generation sequencing or new generation flow technology for MRD detection at the 10^−5^ or 10^−6^ sensitivity level and this led to the inclusion of only seven studies in MRD negativity analysis. The pooled rate of MRD negativity was 97.8% (95%CI 0.935–1.022) and heterogeneity among the studies was low (I^2^ = 0.0%, *p* = .556) (Figure 2(C)). Therefore, the fixed-effects model was used for analysis of MRD negativity.

#### 3.2.2. Subgroup analysis

Next, we performed meta-regression to explore the relevant factors that might influence the efficacy of CAR T therapy including CAR structures, median age of patients, median lines of prior regimens and dose of infused CAR + T cells. No independent factors were found and the smallest *p*-value was .208 in CAR structures (See Supplemental Material for details). We therefore conducted a subgroup analysis based on CAR structures.

**Figure 3.**
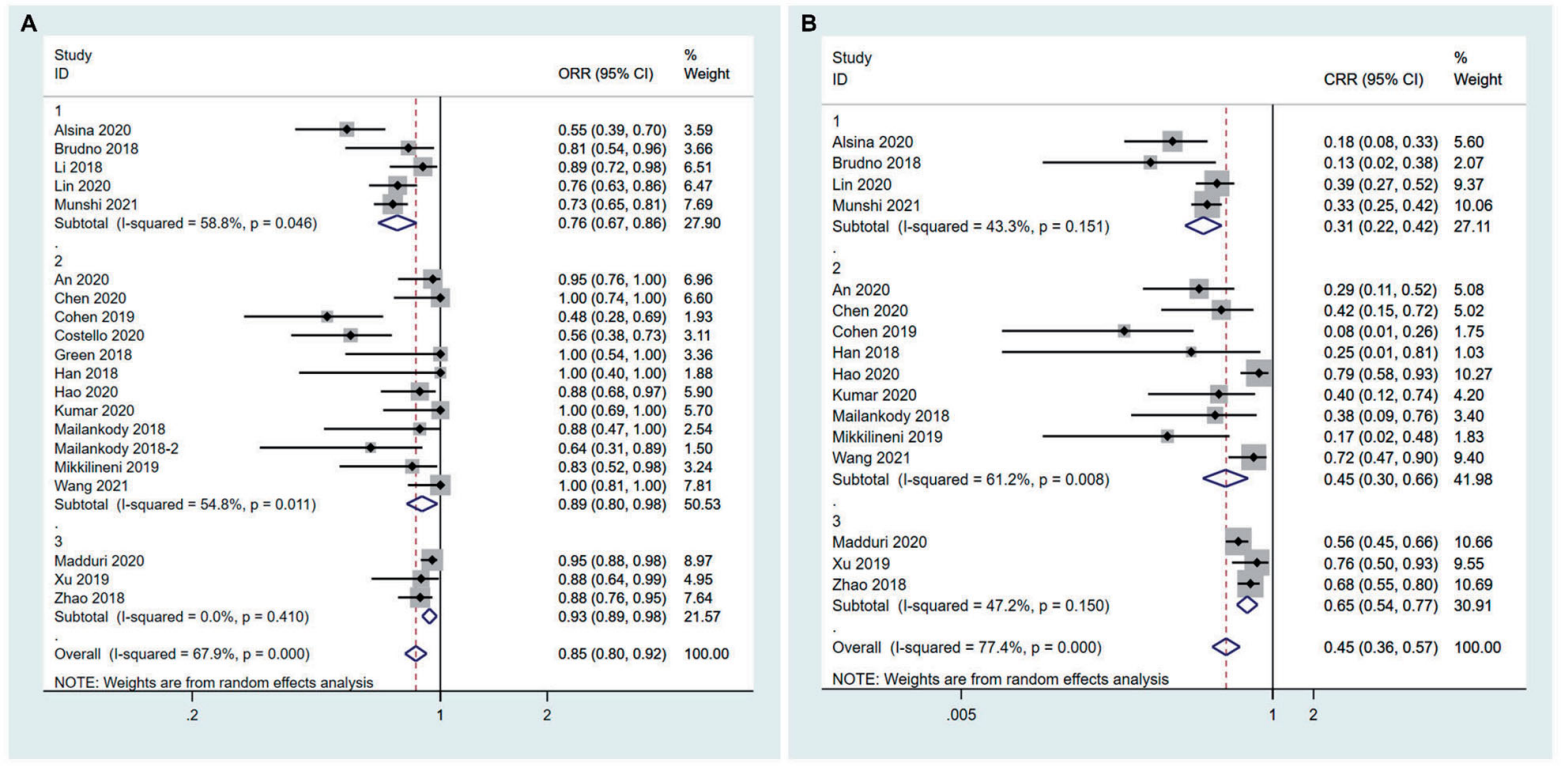
Subgroup analysis of ORR according to the structures of CAR. 1- murine group; 2- human group; 3- dual epitope-binding group. (A) Dual epitope-binding group shows the highest ORR, followed by human group, while the worst ORR is murine group. (B) The CRR in dual epitope-binding group is highest, followed by human group, and murine group has the lowest.

Studies using LCAR-B38M were divided into dual epitope-binding group because LCAR-B38M had two binding domains in one CAR. Other CAR T products with single epitope were further classified into murine group and human group based on their single-chain variable fragment (scFv) species. There were significant differences in ORR among these three groups (*z* = 4.43, *P* < .001). Dual epitope-binding group showed the highest ORR (93.2%, 95%CI 0.889–0.977; I^2^ = 0.0%, *p* = .410), followed by human group (88.8%, 95%CI 0.804–0.981; I^2^ = 54.8%, *p* = .011), while the worst ORR was murine group (75.7%, 95%CI 0.668–0.857; I^2^ = 58.8%, *p* = .046) (Figure 3(A)). The CRR in each group also had significant differences as well (*z* = 6.64,  *P*< .001). The CRR in dual epitope-binding group was highest (64.8%, 95%CI 0.543–0.772; I^2^ = 47.2%, *p* = .150), followed by human group (44.7%, 95%CI 0.304–0.657; I^2^ = 61.2%, *p* = .008), and murine group had the lowest (30.7%, 95%CI 0.225–0.419; I^2^ = 43.3%, *p* = .151) (Figure 3(B)).

We were not able to conduct further subgroup analysis of CRR/ORR in dual epitope-binding group or murine group because of the relatively small number of studies. Human group were further divided into a high dose group (median dose of infused CAR + T cells ≥100 × 10^6^ or ≥1.6 × 10^6^/kg) and a low dose group ( < 100 × 10^6^ or 1.6 × 10^6^/kg) to explore the association of dose and ORR. Similarly, human group studies were divided into a younger group (<60 years old) and an elderly group (≥60 years old) according to the median age of participants. Lastly, based on the median lines of prior regimens, we defined multiply relapsed group (≥6) and relapsed group ( < 6) to conduct subgroup analysis in human group.

**Figure 4.**
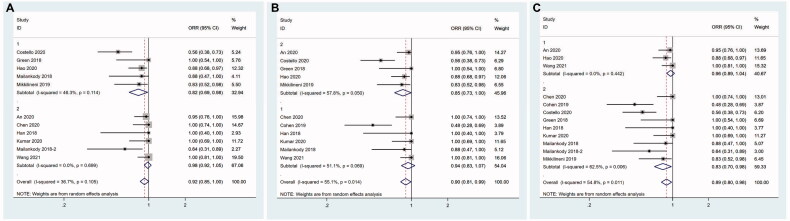
Subgroup analysis of ORR in human group. (A) High dose group has a higher ORR compared with low dose group. 1- low dose group; 2-high dose group. (B) Elderly group has lower ORR compared with younger group. 1-younger group; 2-elderly group. (C) Relapsed group has greater ORR than multiply relapsed group. 1-relapsed group; 2-multiply relapsed group.

We found in studies with human CAR T cells, high dose group had a higher ORR (98.1%, 95%CI 0.918–1.049; I^2^ = 0.0%, *p* = .699) compared with low dose group (82.2%, 95%CI 0.689–981; I^2^ = 46.3%, *p* = 0.114) (*z* = 1.92, *P*= .05) (Figure 4(A)). Elderly group had lower ORR (85.1%, 95%CI 0.725–1.000; I^2^ = 57.8%, *p* = .050) compared with younger group (94.0%, 95%CI 0.825–1.070; I^2^ = 51.1%, *p* = .069) (*z* = 2.11, *P* = .035) (Figure 4(B)). Compared with multiply relapsed group, relapsed group had greater ORR (96.2%, 95%CI 0.892–1.037; I^2^ = 0.0%, *p* = .442 *vs* 82.8%, 95%CI 0.702–0.977; I^2^ = 62.5%, *p* = 0.006) (*z* = 2.33, *P* = .02) (Figure 4(C)).

#### 3.2.3. Binary outcomes

**Figure 5.**
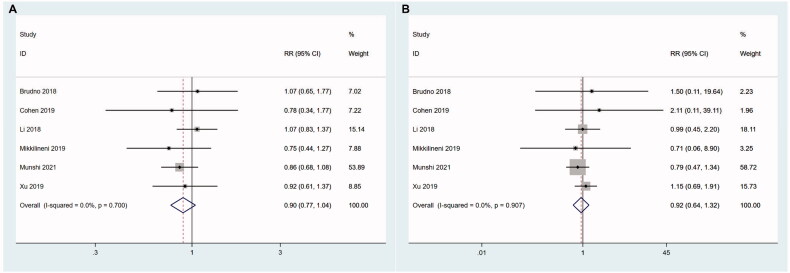
The effects of high-risk karyotypes on the efficacy of CAR T therapy. The results show that there is no significant difference in ORR (A) and CRR (B) between high-risk group and low-risk group.

FISH results of patients were described in detail in 6 literatures. The patients with at least one high-risk cytogenetic feature [17p-, t(4;14), t(14;16)] were defined as high-risk group, while the patients with no high-risk cytogenetic features were defined as low-risk group. We evaluated the effects of high-risk karyotypes on the efficacy of CAR T therapy in the 6 studies. The results showed that there was no significant difference in ORR (*z* = 1.41, *P* = .159; I^2^ = 0.0%, *p* = .700) and CRR (*z* = 0.45, *p* = .656; I^2^ = 0.0%, *p* = .907) between the two groups (Figure 5).

**Figure 6.**
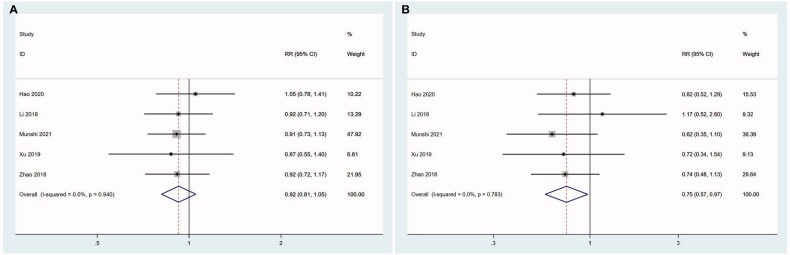
The effects of extramedullary disease (EMD) on the efficacy of CAR T therapy. (A) The results show that there is no significant difference in ORR between EMD group and non-EMD group. In EMD. (B) In EMD group, less patients can achieve CR than non-EMD group.

There were five studies specified whether patients had EMD or not. The patients with EMD were defined as EMD group, while the patients with no EMD were defined as non-EMD group. No significant difference was detected in ORR between these two groups (*z* = 1.19, *P* = .236; I^2^ = 0.0%, *p* = .940). However, CRR in EMD group was lower than non-EMD group (*z* = 2.16, *P*= .03; I^2^ = 0.0%, *p* = .783) (Figure 6).

**Figure 7.**
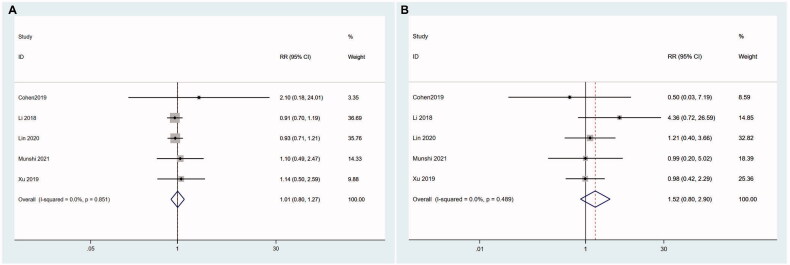
The effects of BCMA expression on the efficacy of CAR T therapy. The results show that there is no significant difference in ORR (A) and CRR (B) between high-expression group and low-expression group.

Patients were divided into two groups (high-expression group and low-expression group) based on BCMA expression on tumour cells in five studies. The threshold for BCMA expression in the first three articles was 50%. We found ORR (*z* = 0.06, *P* = .955; I^2^ = 0.0%, *p* = .851) and CRR (*z* = 1.27,  *P* = .204; I^2^=0%, *p* = .489) were not affected by BCMA expression levels (Figure 7).

### 3.3. Safety

#### 3.3.1. Pooled rates

**Figure 8.**
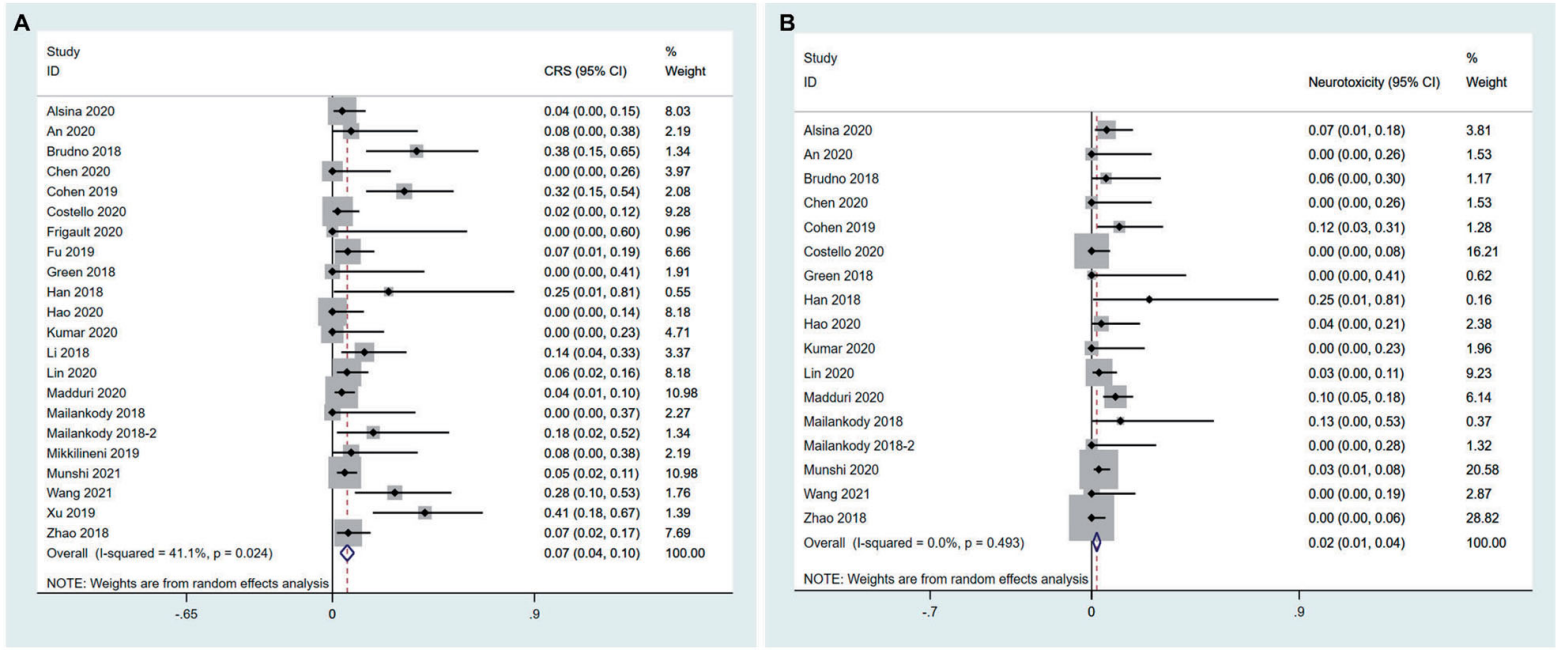
Pooled incidence of CRS and neurotoxicity. (A) The pooled incidence of grade 3–4 CRS is 6.6%. (B) The pooled incidence of grade 3–4 neurotoxicity is 2.2%.

The incidences of grade 3–4 CRS were described in all the 22 articles and the incidences of grade 3–4 neurotoxicity caused by CAR T cell infusion were found in 17 articles. The pooled incidence of grade 3–4 CRS was 6.6% (95%CI 0.036–0.096) with moderate evidence of heterogeneity across the studies (I^2^ = 41.1%, *p* = .024) (Figure 8(A)). The pooled incidence of grade 3–4 neurotoxicity was 2.2% (95%CI 0.006–0.038) and heterogeneity was not observed among the studies (I^2^ = 0.0%, *p* = .493) (Figure 8(B)).

#### 3.3.2. Subgroup analysis

**Figure 9.**
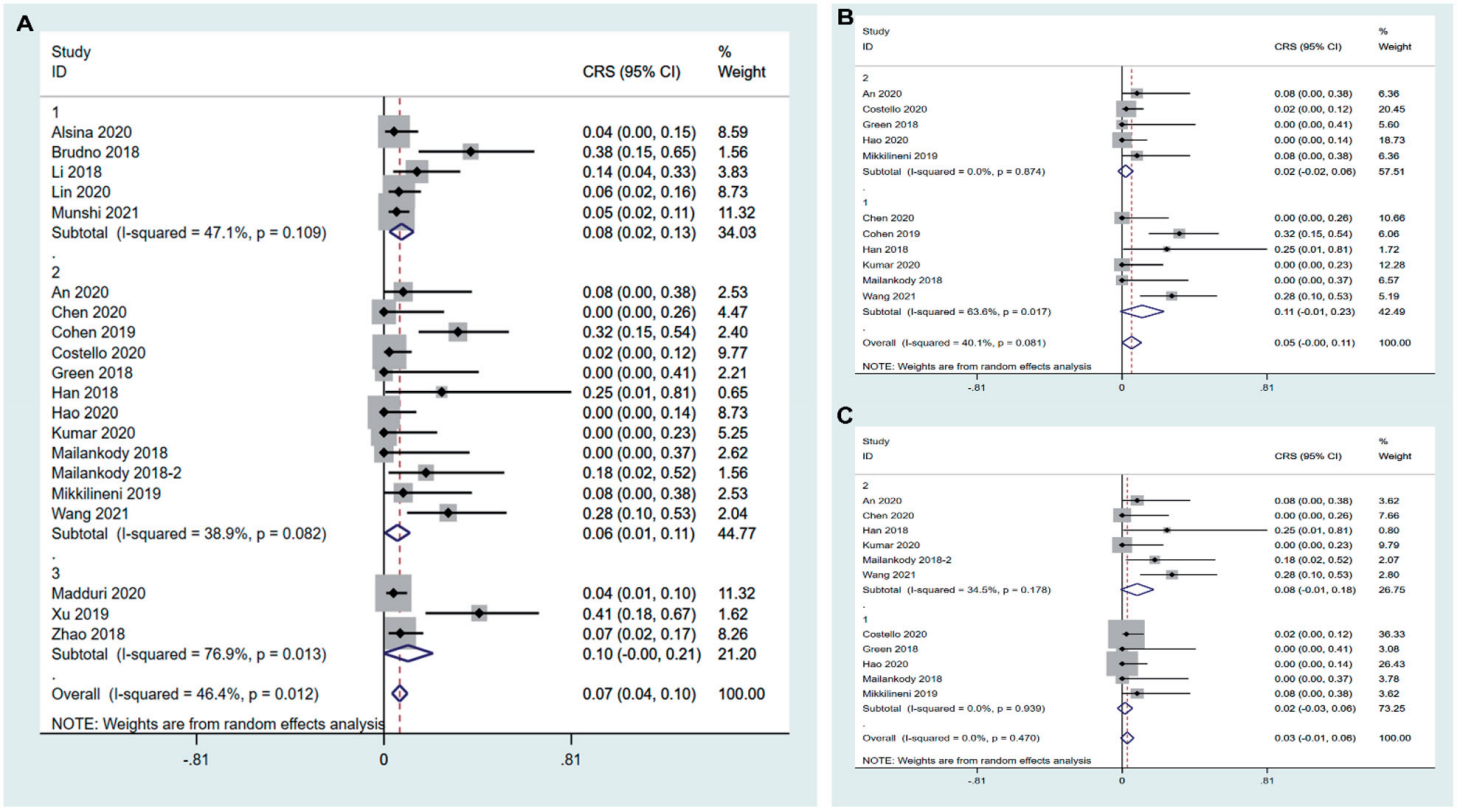
Subgroup analysis of incidence of grade 3–4 CRS. (A) Human group shows significantly reduced incidence of grade 3–4 CRS, followed by murine group and dual epitope-binding group has the highest incidence of grade 3–4 CRS. 1-murine group; 2-human group; 3-dual epitope-binding group. (B) In the studies using humanized CAR T cells, elderly group has a lower incidence of grade 3–4 CRS compared with younger group. 1-younger group; 2-elderly group. (C) In the studies using humanised CAR T cells, high dose group shows a trend to have higher incidence of grade 3–4 CRS than low dose group without reaching a statistical significance. 1-low dose group; 2-high dose group.

We did not perform subgroup of grade 3–4 neurotoxicity due to the small number of studies. Considering the incidence of grade 3–4 CRS was probably associated with structure of CAR T cells, dose of infused CAR^+^ T cells and median age of patients, we carried out subgroup analysis for these three factors. Human group showed significantly reduced incidence of grade 3–4 CRS (5.9%, 95%CI 0.005–0.112; I^2^ = 38.9%, *p* = .082), followed by murine group (7.6%, 95%CI 0.024–0.129; I^2^ = 47.1%, *p* = .109) and dual epitope-binding group had the highest (10.4%, 95%CI −0.003–0.212) incidence of grade 3–4 CRS (*z* = 4.04,  *P*< .001) (Figure 9(A)).

Similar to the ORR above, we conducted further subgroup analysis in human group with dose of infused CAR^+^ T cells and median age of patients. Rather unexpected, the elderly group had a lower incidence of grade 3–4 CRS (2.0%, 95%CI −0.022–0.062; I^2^ = 0.0%, *p* = .874) compared with younger group (11.2%, 95%CI −0.001–0.108; I^2^ = 63.6%, *p* = .017) (*z* = 1.92, *P* = .05) (Figure 9(B)). High dose group showed a trend to have higher incidence of grade 3–4 CRS than low dose group without reaching a statistical significance (8.4%, 95%CI −0.009–0.177; I^2^ = 34.5%, *p* = 0.178 vs 1.6%, 95%CI −0.026–0.058; I^2^ = 0.0%, *p* = .939) (*z* = 1.53, *P* = .127) (Figure 9(C)).

### 3.4. Survival analysis

**Figure 10.**
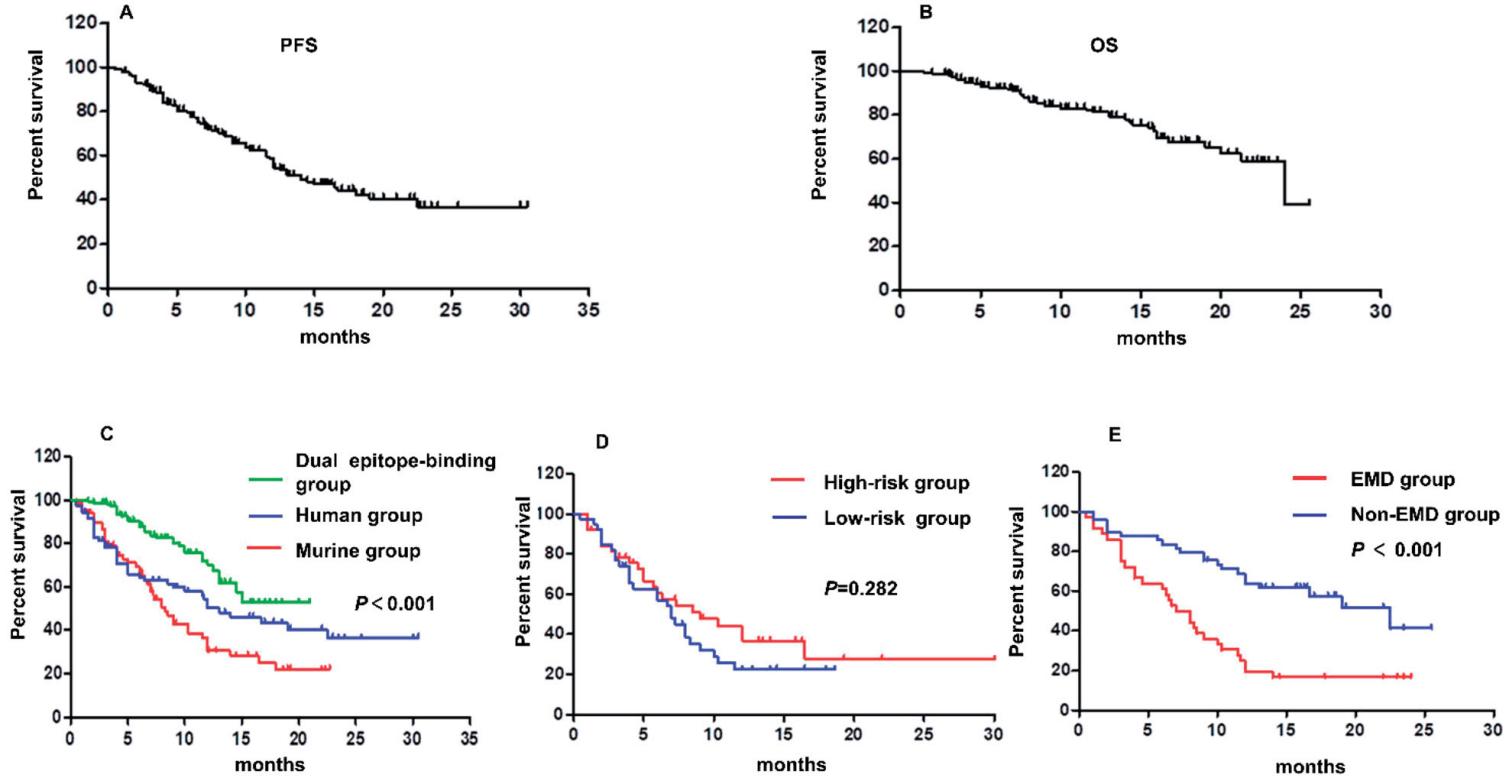
Survival analysis of CAR T therapy. (A) The median PFS is 14.5 months for R/R MM patients who receive CAR T therapy. One-year PFS is 54.5% and two-year PFS is 36.9%. (B) The median OS is 24 months, and the estimated 1-year OS is 81.2% and two-year OS is 39.1%. (C) The longest PFS is observed in dual epitope-binding group, followed by human group and murine group has the shortest PFS. (D) No significant difference in PFS is identified between the high-risk and low-risk group. (E) The PFS of non-EMD group is superior to the PFS of EMD group.

PFS data was extracted from 10 studies including 294 patients. The median PFS was 14.0 months, and the Kaplan-Meier estimate of one-year PFS was 54.5% and two-year PFS was 36.9%. OS data was collected from seven studies including 251 patients. Median OS was 24 months, and the estimated one-year OS was 81.2% and two-year OS was 39.1% (Figure 10).

There were insufficient data to undertake subgroup analysis of OS, so we only carried out subgroup analysis of PFS (Figure 10). There were 71 patients in human group, 67 patients in murine group and 157 patients in dual epitope-binding group. The longest PFS was observed in dual epitope-binding group, followed by human group and murine group had the shortest PFS (*P* < .001).The results of FISH were available for 77 patients, and 38 patients in high-risk group while 39 patients in low-risk group. No significant difference in PFS was identified between the two groups (*P* = .282). There were 36 patients in EMD group and 49 patients in non-EMD group. The PFS of non-EMD group was superior to the PFS of EMD group (*P* < .001). PFS data were available for only seven patients in low-expression group of BCMA. As a result, survival analysis was not performed to evaluate the effect of BCMA expression level on patients’ survival.

### 3.5. Sensitivity analysis and publication bias assessment

**Figure 11.**
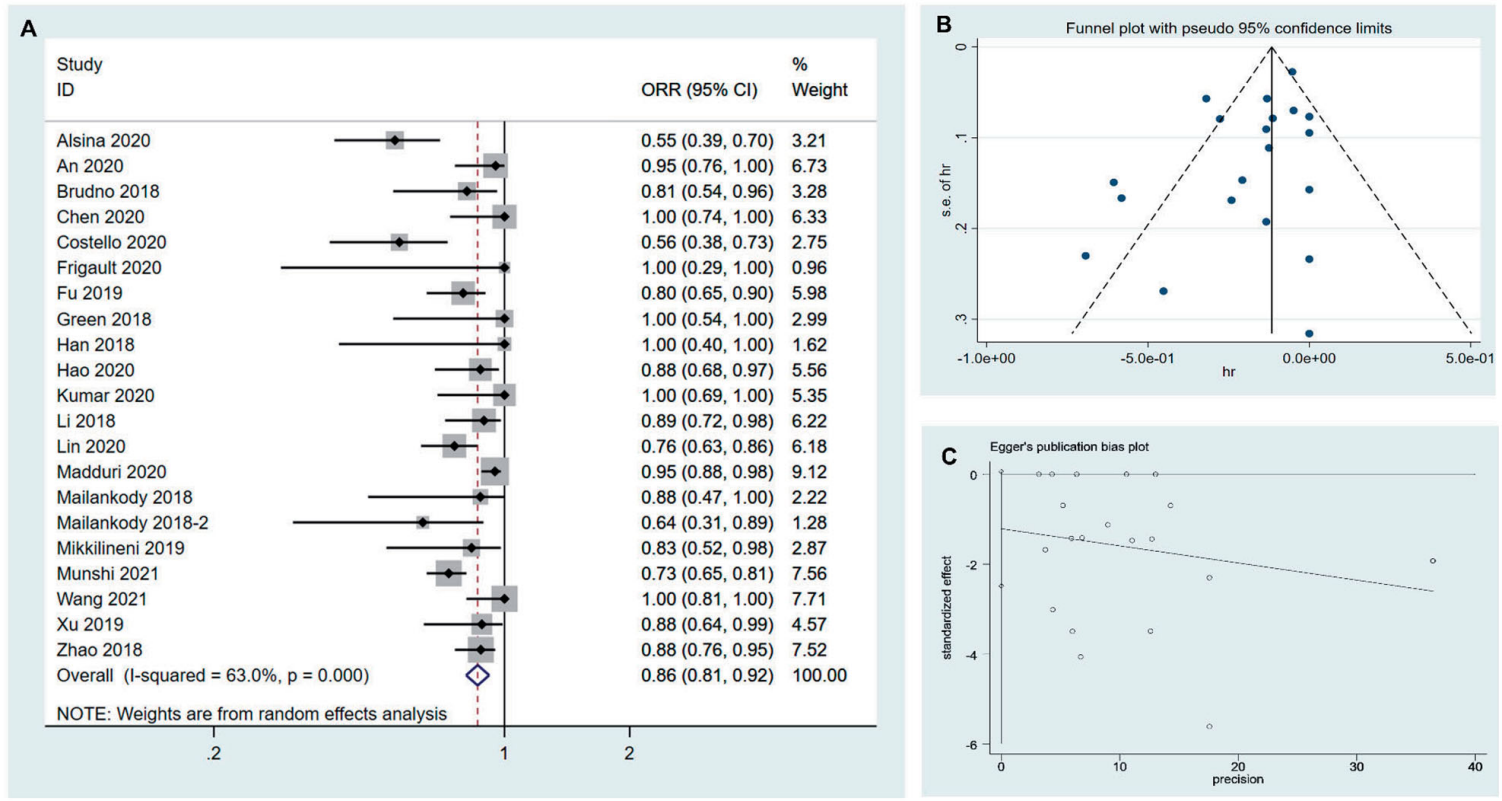
Sensitivity analysis and publication bias assessment. (A) Sensitivity analysis is performed by the “leave-one-out” approach to assess the stability of our results. (B) No significant publication bias is noted on funnel plots. (C) The results of Egger’s test reveal no evidence of publication bias.

Sensitivity analysis was performed by the “leave-one-out” approach to assess the stability of our results. As shown in Figure 11(A), removal of one study every time from pooled analysis did not change ORR significantly. The pooled ORR was 86.2% (95%CI 0.809–0.919; I^2^ = 63.0%, *p* < .001). No significant publication bias was noted on funnel plots (Figure 11(B)). The results of Egger’s test (*p* = .061, 95%CI −2.485–0.061) and Begg’s test (z= −1.33, *p* = .184) revealed no evidence of publication bias (Figure 11(C)).

## 4. Discussion

The first-in-human clinical trial on anti-BCMA CAR T therapy for R/R MM was reported by Brudno [39] from the National Cancer Institute. In the high dose group, ORR was 81.3% and it was very encouraging in heavily pre-treated patients with MM. Meanwhile, this study suggested that higher dose of infused CAR^+^ T cells could bring better remission and it was consistent with the results of our subgroup analysis. Our data also showed that infusion with a higher dose of CAR T cells might increase the incidence of severe CRS, suggesting it was not feasible to enhance the therapeutic effect by simply increasing the dose. Therefore, further work still needs to be done.

Subsequently, the number of studies on anti-BCMA CAR T therapy is rapidly growing. Our meta-analysis demonstrated that the pooled ORR was 85.2%, with the pooled CRR 47.0%. The pooled rate of MRD negativity even reached up to 97.8%. Notably, CAR T showed a better effective rate in R/R MM than some other treatment regimens including daratumumab/bortezomib/dexamethasone (ORR was 82.9%) [40], ixazomib/lenalidomide/dexamethasone (ORR was 78%) [41] and carfilzomib/dexamethasone (ORR was 77%) [42]. The heterogeneity of ORR and CRR among studies might be related to the difference of CAR T cells and enrolled patients. Therefore, heterogeneity was improved by doing subgroup analysis. Further, survival analysis results showed that the median PFS of CAR T therapy was 14.0 months and median OS was 24 months. All above data implied that anti-BCMA CAR T therapy is an effective and durable treatment that is able to provide deep remission for patients with R/R MM. Its benefits are apparent for only one infusion would improve patients’ disease-related quality of life over such a long time period.

Another field we paid attention to was the AEs of anti-BCMA CAR T cell infusion, which to our knowledge, mostly presented as CRS and neurotoxicity [14]. The pooled incidence of grade 3–4 CRS was 6.6% and neurotoxicity was 2.2%. With prompt recognition and anti-IL-6 treatment, the therapy-related mortality of CAR T is extremely low in fact [4]. There were some concerns about the safety of CAR T therapy among elderly patients due to their poor tolerance. Our study indicated that severe CRS occurred less frequently in elderly patients by using humanized CAR T cells. An explanation of this phenomenon is currently not available. We can only speculate that elderly patients might have lower immunity and less cytokine releasing. Taken together, our results showed anti-BCMA CAR T is a safe treatment even in elderly patients.

It is worth further investigation to decide which patients are suitable candidates for anti-BCMA CAR T therapy for this treatment will be widely used in the near future. In many clinical trials, baseline BCMA positivity on tumour cells is an inclusion criterion. However, the threshold of BCMA expression were varied in different studies and patients’ outcomes were reported not correlated with the levels of BCMA expression [43]. We included five studies with 196 patients and found whether the BCMA expression of tumour cells was greater than 50% did not affect ORR and CRR which was consistent with most of the reports. A possible reason is that these CAR T products had a high BCMA binding affinity and tumour cells are eliminated even with little amount of binding with CAR T cells. Thus, the use of CAR T therapy should not be limited by baseline BCMA expression. Moreover, high-risk cytogenetic features and EMD are two established risk factors of poor prognosis in MM [1,44]. In this study, we contrasted the efficacy of CAR T therapy in patients with and without poor prognosis factors. We were pleasantly surprised that the ORR, CRR and PFS showed no significant difference between patients with and without high-risk cytogenetic features. These results suggest that poor outcomes associated with high-risk karyotypes in MM patients could be overcome by CAR T infusion. The remission rate (ORR) was similar between patients with and without EMD, while the PFS and CRR of patients with EMD was significantly shorter and lower than patients without EMD. This indicates that EMD is associated with increased risk of recurrent and less “deep remission” after CAR T cell infusion and better treatments for EMD are still badly needed.

Currently, CAR T therapy is carried out in patients experiencing multiple relapses while its use in newly diagnosed patients is only seen in case reports. However, the optimal timing for initiation of CAR T cell infusion is unclear. After the subgroup analysis of median lines of prior regimens, we found that the ORR of CAR T therapy decreased when patients received more than or equal to six lines of prior regimens. Data from clinical trials confirmed that the presence of naïve T cells, stem memory T cells and central memory T cells in the premanufacture product is related to the clinical response of MM patients [21]. Das et al. [45] found that cumulative chemotherapy cycles depleted naïve T cells in many pediatric cancers which illustrated repeated chemotherapy could hamper T-cell fitness for CAR T cell manufacture. So, we recommend to use CAR T cell infusion as earlier lines of treatment for patients with R/R MM.

As the number of clinical trials continues to increase, researchers are exploring the optimal structure of CAR T cells. Generally, CAR consists of an extracellular antigen-recognition domain (scFv), a transmembrane domain, and an intracellular domain including co-stimulation and signalling components [6]. The scFv on earliest-appearing CAR T cells was from murine [39]. Human-derived-scFv and llama-derived (dual epitope-binding) CAR T products occurred later. We conducted a subgroup analysis according to the structure of CAR, and the results showed LCAR-B38M (llama-derived, dual epitope-binding) offered the most favorable efficacy. However, the incidence of CRS caused by LCAR-B38M was significantly higher which limited its application prospect. CAR T cells with humanized scFv had a higher remission rates (ORR and CRR) and lowest incidence of CRS. The murine-derived CAR T cells exhibited relatively poor safety and efficacy. However, there are two trials [29,39] in murine group using CD28 co-stimulatory molecule. Existing studies show CAR T cells with a 4-1BB costimulatory domain are observed to persist longer than CAR T cells with a CD28 costimulatory domain [46]. This might be one of the reasons for lower efficacy of murine- derived CAR T cells. So, results from our comprehensive analyses showed humanized CAR T cells were superior to LCAR-B38M and murine-derived CAR T cells at present.

We acknowledge that there were limitations in this study. First, the number of included studies was relatively limited. Also, there were some unreported outcomes. A random-effects model was used to minimize the influence of heterogeneity, and we carried out subgroup analysis to explore the reasons for the heterogeneity.

In conclusion, anti-BCMA CAR T therapy is effective and safe for patients with R/R MM. It can improve the prognosis of patients with high-risk cytogenetic features and can be well tolerated in the elderly. Patients are likely to benefit from an earlier use of CAR T therapy at the time of relapse. Moreover, CAR T cells with humanized scFv have obvious advantages based on the existing data. Further studies with large sample size and are expected to confirm our results.

## Supplementary Material

Supplemental Material

## Data Availability

The data of this study are available from the corresponding author upon request.
