# Supplementary material for: Comprehensive meta-analysis of anti-BCMA chimeric antigen receptor T-cell therapy in relapsed or refractory multiple myeloma
Source: Ann Med. 2021 Aug 30;53(1):1547–59. doi: 10.1080/07853890.2021.1970218 (PMC8409966; doi:10.1080/07853890.2021.1970218)
Supplement: Supplemental Material [file IANN_A_1970218_SM8286.docx]

1. **Retrieval strategy of PubMed**

1.1 "Immunotherapy, Adoptive"[Mesh]

1.2 (((((((Chimeric Antigen Receptor Therapy[Title/Abstract]) OR (CAR T-Cell Therapy[Title/Abstract])) OR (CAR T Cell Therapy[Title/Abstract])) OR (CAR T-Cell Therapies[Title/Abstract])) OR (T-Cell Therapies, CAR[Title/Abstract])) OR (T-Cell Therapy, CAR[Title/Abstract])) OR (Therapies, CAR T-Cell[Title/Abstract])) OR (Therapy, CAR T-Cell[Title/Abstract])

1.3 #1 OR #2

1.4 "Multiple Myeloma"[Mesh]

1.5 ((((((((((((((((((Multiple Myelomas[Title/Abstract]) OR (Myeloma*, Multiple[Title/Abstract])) OR (Myeloma, Plasma-Cell[Title/Abstract])) OR (Myeloma, Plasma Cell[Title/Abstract])) OR (Myelomas, Plasma-Cell[Title/Abstract])) OR (Plasma-Cell Myeloma[Title/Abstract])) OR (Plasma-Cell Myelomas[Title/Abstract])) OR (Myelomatosis[Title/Abstract])) OR (Myelomatoses[Title/Abstract])) OR (Plasma Cell Myeloma[Title/Abstract])) OR (Cell Myeloma, Plasma[Title/Abstract])) OR (Cell Myelomas, Plasma[Title/Abstract])) OR (Myelomas, Plasma Cell[Title/Abstract])) OR (Plasma Cell Myelomas[Title/Abstract])) OR (Kahler Disease[Title/Abstract])) OR (Disease, Kahler[Title/Abstract])) OR (Myeloma-Multiple[Title/Abstract])) OR (Myeloma Multiple[Title/Abstract])) OR (Myeloma-Multiples[Title/Abstract])

1.6 #4 OR #5

1.7 (clinical[Title/Abstract] AND trial[Title/Abstract]) OR clinical trials as topic[MeSH Terms] OR clinical trial[Publication Type] OR random*[Title/Abstract] OR random allocation[MeSH Terms] OR therapeutic use[MeSH Subheading]

1.8 #7 AND #6 AND #3

1. Results of meta-regression of overall response rate.

| **Variables** | ***P* value** |
| --- | --- |
| Structure 1 | 0.208 |
| Structure 2 | 0.264 |
| Structure 3 | 0.409 |
| Line 1 | 0.666 |
| Line 2 | 0.264 |
| Year 1 | 0.508 |
| Year 2 | 0.508 |
| Dose 1 | 0.270 |
| Dose 2 | 0.753 |
